# A randomized and blinded comparison of qPCR and NGS-based detection of aneuploidy in a cell line mixture model of blastocyst biopsy mosaicism

**DOI:** 10.1007/s10815-016-0784-3

**Published:** 2016-08-06

**Authors:** David Goodrich, Xin Tao, Chelsea Bohrer, Agnieszka Lonczak, Tongji Xing, Rebekah Zimmerman, Yiping Zhan, Richard T. Scott Jr, Nathan R. Treff

**Affiliations:** 1 Reproductive Medicine Associates of New Jersey, 140 Allen Rd, Basking Ridge, NJ 07920 USA; 2 Foundation for Embryonic Competence Inc, 140 Allen Rd, Suite 300, Basking Ridge, NJ 07920 USA

**Keywords:** Comprehensive chromosome screening, Whole genome amplification, Next-generation sequencing, Quantiative real time PCR, Mosaicism

## Abstract

**Purpose:**

A subset of preimplantation stage embryos may possess mosaicism of chromosomal constitution, representing a possible limitation to the clinical predictive value of comprehensive chromosome screening (CCS) from a single biopsy. However, contemporary methods of CCS may be capable of predicting mosaicism in the blastocyst by detecting intermediate levels of aneuploidy within a trophectoderm biopsy. This study evaluates the sensitivity and specificity of aneuploidy detection by two CCS platforms using a cell line mixture model of a mosaic trophectoderm biopsy.

**Methods:**

Four cell lines with known karyotypes were obtained and mixed together at specific ratios of six total cells (0:6, 1:5, 2:4, 3:3, 4:2, 5:1, and 6:0). A female euploid and a male trisomy 18 cell line were used for one set, and a male trisomy 13 and a male trisomy 15 cell line were used for another. Replicates of each mixture were prepared, randomized, and blinded for analysis by one of two CCS platforms (quantitative polymerase chain reaction (qPCR) or VeriSeq next-generation sequencing (NGS)). Sensitivity and specificity of aneuploidy detection at each level of mosaicism was determined and compared between platforms.

**Results:**

With the default settings for each platform, the sensitivity of qPCR and NGS were not statistically different, and 100 % specificity was observed (no false positives) at all levels of mosaicism. However, the use of previously published custom criteria for NGS increased sensitivity but also significantly decreased specificity (33 % false-positive prediction of aneuploidy).

**Conclusions:**

By demonstrating increased false-positive diagnoses when reducing the stringency of predicting an abnormality, these data illustrate the importance of preclinical evaluation of new testing paradigms before clinical implementation.

## Introduction

Comprehensive methods for preimplantation aneuploidy screening have become a common part of infertility care. The use of preimplantation screening (PGS) and the ability to diagnose aneuploidy of all 24 human chromosomes and improvement in the accuracy of amplification strategies represent important advancements that have clearly improved outcomes when applied to embryo selection strategies for patients undergoing IVF treatment [1–4]. The observed improvements in clinical outcomes are based on the simple fact that approximately one third of human preimplantation embryos are chromosomally abnormal. It is well established that embryonic aneuploidy rates increase dramatically with advanced maternal age. However, in some cases, post-fertilization mitotic errors in chromosome segregation have been observed [5]. These errors lead to chromosomal mosaicism within the developing embryo and represent a complex diagnostic challenge. For example, most trophectoderm biopsies contain between five and eight cells, a relatively small proportion of a blastocyst-stage embryo which contains a large number of cells [6–9].

Comprehensive chromosome screening (CCS) platforms generally quantify chromosome copy number and predict aneuploidy when the relative copy numbers reach a specific threshold for diagnosing a gain or loss [10]. Recent research has suggested that intermediate levels of gains or losses from array comparative genomic hybridization (aCGH) data are indicative of mosaicism and predictive of reduced reproductive potential of the remaining embryo. Given that 26–33 % of embryos predicted to have mosaic aneuploidy led to successful deliveries, more careful consideration for not only biological phenomenon, but also false-positive diagnoses should be given [11, 12].

Some have argued that next-generation sequencing (NGS)-based strategies may provide an enhanced and unique opportunity to predict mosaicism within a trophectoderm biopsy [13], and one group recently published criteria for predicting mosaicism within a trophectoderm biopsy using intermediate values of copy number [14]. Nonetheless, there remains a lack of data regarding the actual capabilities and comparative performance of contemporary CCS platforms for predicting aneuploidy in a mosaic sample. Previous studies have used cell lines and whole genome amplification (WGA) products to create mixtures of euploid and aneuploid cells as a model for a mosaic trophectoderm biopsy [7, 15]. However, these prior studies used either SNP array or array (aCGH), which are becoming less utilized with the introduction of more cost effective approaches, and were limited by a small sample size at each mixture level. This study evaluated two commercially available CCS platforms, involving quantitative polymerase chain reaction (qPCR) and NGS, for their sensitivity and specificity of aneuploidy detection in a cell line mixture model of a mosaic trophectoderm biopsy.

## Materials and methods

**Fig. 1:**
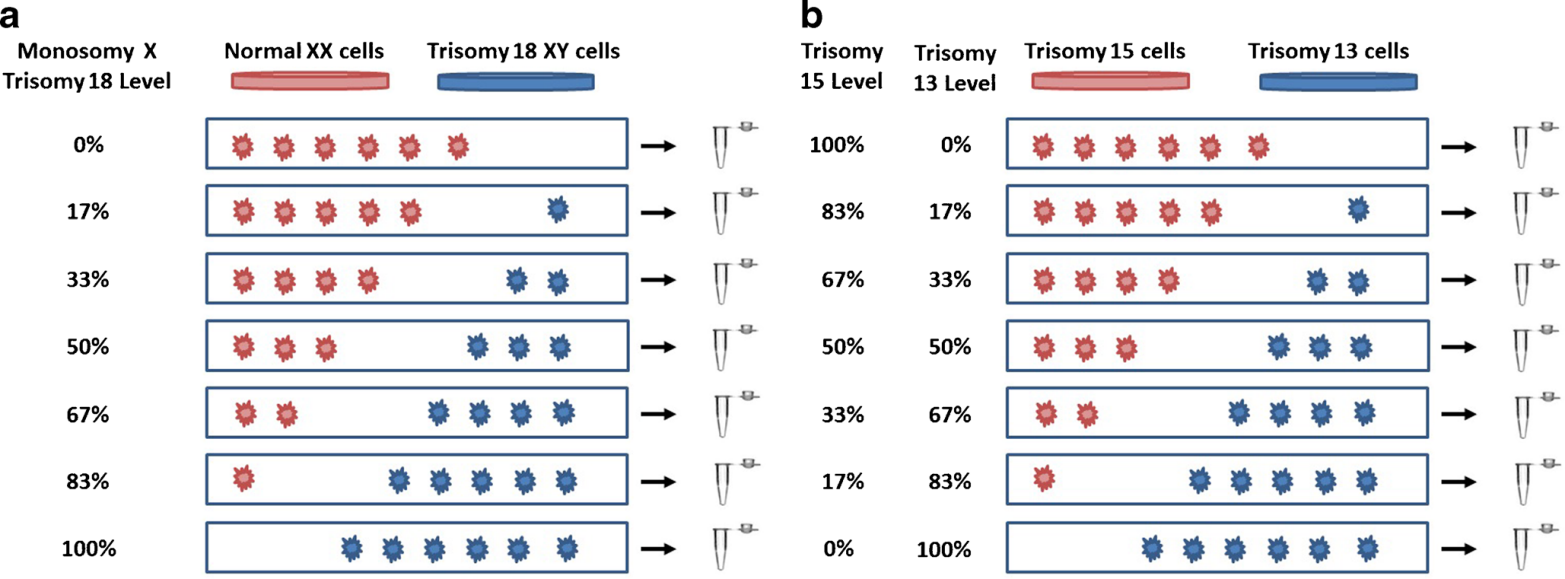
**a** Mixture model experimental strategy illustration for preparation of samples involving a male trisomy 18 cell line and a female euploid cell line where increasing levels of trisomy 18 and monosomy X are expected and **b** trisomy 15 cells mixed with trisomy 13 cells where inverse changes in levels of each aneuploidy are expected. Cells are mixed in a single tube in known ratios of six total cells (0:6, 1:5, 2:4, 3:3, 4:2, 5:1, and 6:0) to mimic various levels of mosaicism in a trophectoderm biopsy

In order to establish positive controls for specific levels of mosaicism, four adult human fibroblast cell lines, GM00321 (46,XX), GM01359 (47,XY,+18), GM03184 (47,XY,+15), and GM02948 (47,XY,+13), were purchased from the Coriell Cell Repository (Camden, NJ). Each cell line was previously characterized for karyotypes by the supplier. The cells were cultured and passaged once prior to collection as recommended. Individual cells were obtained under a dissecting microscope and mixed together at specific ratios of six total cells (0:6, 1:5, 2:4, 3:3, 4:2, 5:1, and 6:0). The euploid female and trisomy 18 male cell lines were used for one set of mixtures (Fig. 1a), and the trisomy 13 and trisomy 15 cell lines were used for another set of mixtures (Fig. 1b). Twelve replicates of each mixture level were collected and then divided equally and randomly to one of two CCS platforms for analysis. One protocol involved the use of either SelectCCS (Foundation for Embryonic Competence Inc., Basking Ridge, New Jersey), a previously validated qPCR platform [2, 3, 16], or VeriSeq PGS (Illumina Inc., Santa Clara, CA), a commercially available method involving WGA and next-generation sequencing (NGS) on a MiSeq. Blinded computational prediction of aneuploidy was made with either (i) previously established criteria for qPCR [16], termed “default qPCR,” (ii) as recommended by the supplier utilizing the automatic aneuploidy calls made by Bluefuse Multi software (BlueFuse, Illumina Inc., version 4.2(20289)), termed “default VeriSeq,” or (iii) using previously defined [14] customized criteria for VeriSeq PGS (which examine changes in the median copy numbers and override automated calls made by Bluefuse Multi software), termed “custom VeriSeq”. It is important to note here that the default settings of the software used by these platforms in this study are not able to be manually altered and, any further tweaking of criteria must be done post-analysis with an independent algorithm. Therefore, two platforms were tested using default settings, and one platform (NGS) was further investigated using additional published criteria. As previously defined [13], the custom VeriSeq analysis criteria predicted additional “mosaic” aneuploidies when median copy number values of the chromosomes were either between 1.2 and 1.8 or between 2.2 and 2.8 [14].

After aneuploidy predictions were made, the samples were unblinded and evaluated for consistency with the expected results. Sensitivity was defined as the percentage of samples which were predicted as abnormal for the correct chromosome depending on which mixture set was tested (i.e., trisomy 13, 15, or 18, or monosomy X (the change as chromosome X goes from being female (disomic X) to male (monosomic X); Fig. 1) and was determined for each chromosome (*n* = 24) at each of the seven mixture levels for each platform and analysis setting.. Specificity was defined as the percentage of samples where euploidy was predicted for all the chromosomes expected to be normal or disomy (*n* = 84 for each method: the number of remaining autosomes (21) multiplied by the number of sets of samples (4)). Platform performance was compared using a chi-square test for significance at each mixture level for sensitivity and for overall specificity. For example, the number of qPCR cases in which trisomy 18 was detected or not detected in the 17 % trisomy 18 mixture level samples was compared to the number detected or not detected using VeriSeq at the same mixture level. The same process was used at all mixture levels for each of the four chromosomes (13, 15, 18, and X) and each analysis methods tested.

## Results

**Fig. 2:**
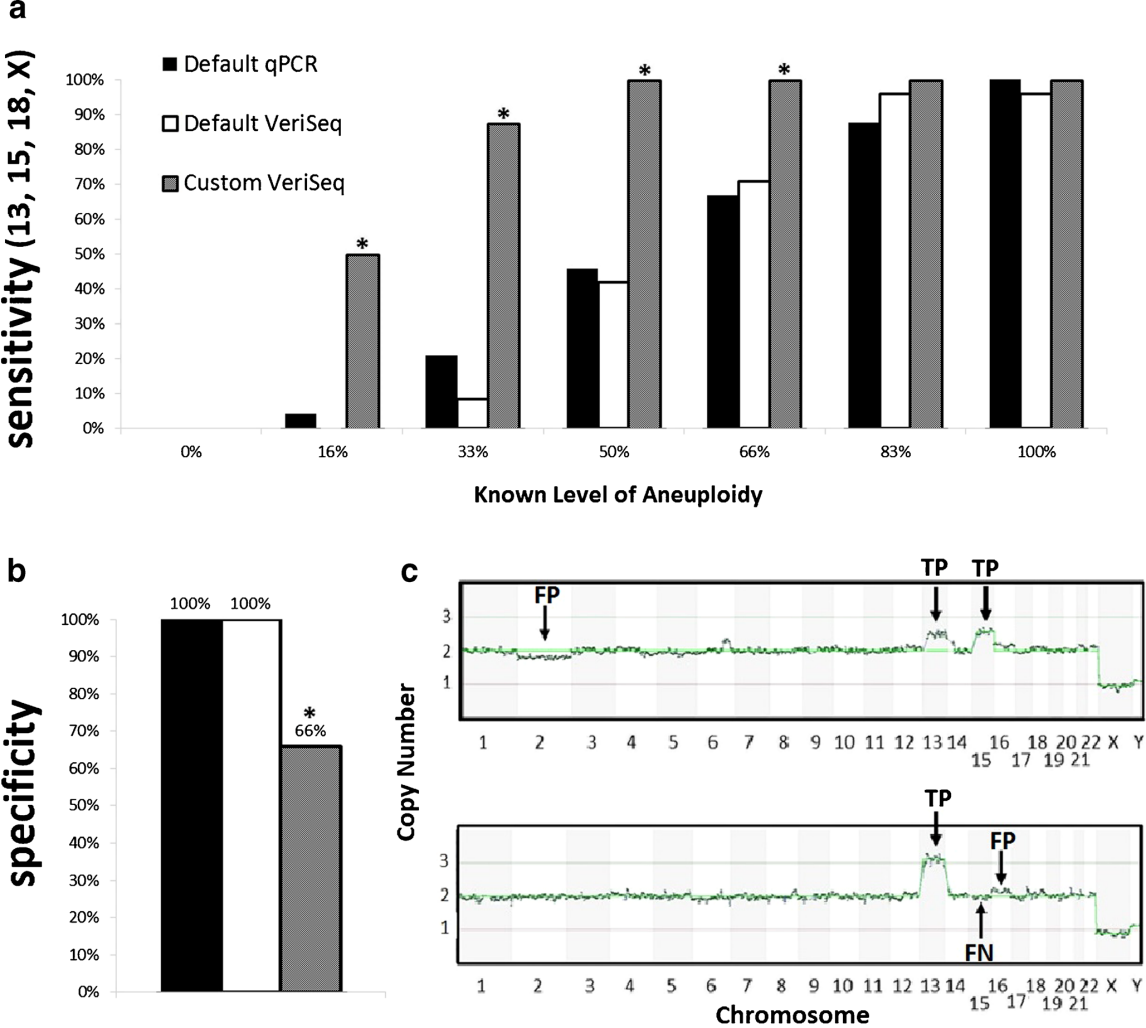
**a** Sensitivity across three sets of analyses for each mixture level: qPCR default settings, VeriSeq default settings, and VeriSeq with criteria defined by Vera-Rodriguez, et al. [14] (custom VeriSeq). Sensitivity is based on detecting trisomy of 13, 15, and 18, and monosomy of X (*n* = 24 at each mixture level for each platform). *Asterisks* indicate statistically significant differences. **b** Specificity across all samples for the same three analysis methods based on the frequency of detecting a normal copy number for each of the remaining chromosomes known to be uniformly normal. *Asterisks* indicate statistically significant differences. **c** Example plots of samples which were given false-positive predictions of mosaic aneuploidy using previously published custom settings for VeriSeq PGS data analysis. *FP* false positive, *TP* true positive, *FN* false negative

Analysis of qPCR and NGS results demonstrated the ability to predict an abnormality correctly in samples containing as little as 17 % aneuploidy. Increased frequency of detection was observed as increasing levels of aneuploidy were present (Fig. 2a). When comparing default qPCR and NGS analysis settings, there was no difference in sensitivity at any mixture level, 0 % *p* = 1, 17 % *p* = 0.312, 33 % *p* = 0.637, 50 % *p* = 0.771, 67 % *p* = 0.756, 83 % *p* = 0.296, and 100 % *p* = 0.312 (Fig. 2a). Overall specificity was equivalent at 100 % for both platforms with default methods of analysis (Fig. 2b). That is, no false-positive predictions of aneuploidy were made by either platform when standard criteria were applied.

In contrast, when applying custom analysis criteria (not using automated calls from Bluefuse Multi software), as defined by Vera-Rodriguez et al. [14], and although significantly improved sensitivity of detecting aneuploidy was observed from 17 to 66 % aneuploidy levels (*p* < 0.05) (Fig. 2a), the gain in sensitivity resulted in a significant increase (*p* < 0.001) in the rate of false positives. The false-positive rate increased from 0 % (0/84), using default qPCR or VeriSeq analysis methods, to 33 % (28/84) with a custom VeriSeq-based analysis (Fig. 2b). These results illustrate the balance between sensitivity and specificity for detecting aneuploidy from intermediate copy number values. Examples of samples which gave inaccurate predictions of aneuploidy are shown in Fig. 2c. The first plot shows a sample with a false positive prediction of mosaic monosomy for chromosome 2. The same sample also shows that the custom criteria gave better sensitivity to detection of trisomy of chromosome 13 (a true positive), where default settings failed. The second example shows a sample with a false-positive prediction of mosaic trisomy of chromosome 16, a true positive for trisomy of chromosome 13 (83 % mixture level), and a false negative for trisomy of chromosome 15 (17 % mixture level). Overall, one third of the samples gave similar false positives when applying previously published custom analysis criteria.

**Fig. 3:**
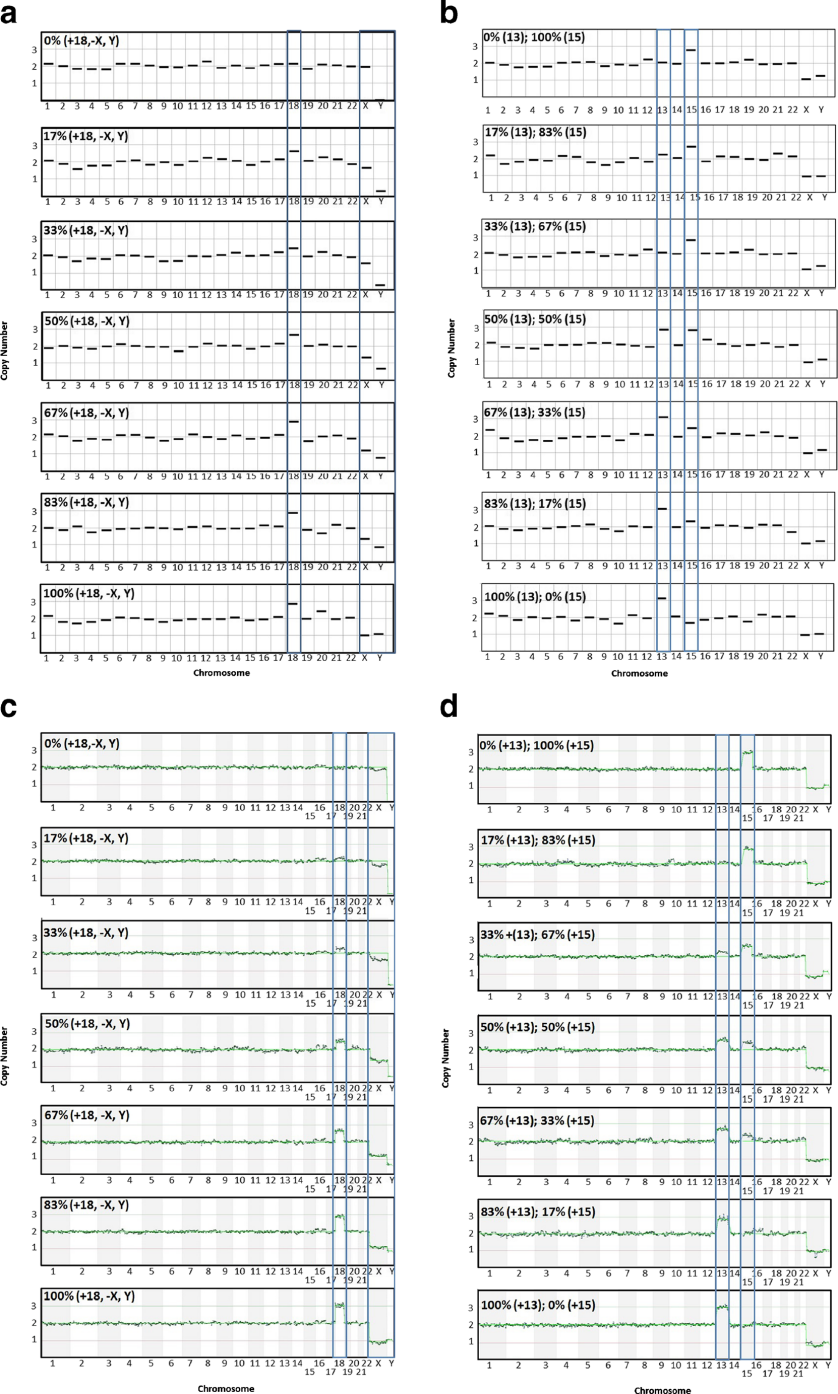
Example plots from qPCR CCS and VeriSeq PGS analyses of the trisomy 18 male and euploid female (**a** and **c**), and trisomy 13 and trisomy 15 (**b** and **d**) six-cell mixture sets. *Vertical boxes* outline chromosomes of interest in each set. As the level of aneuploidy increases in the sample, there is a concomitant change in the copy number values of the chromosomes of interest

**Fig. 4:**
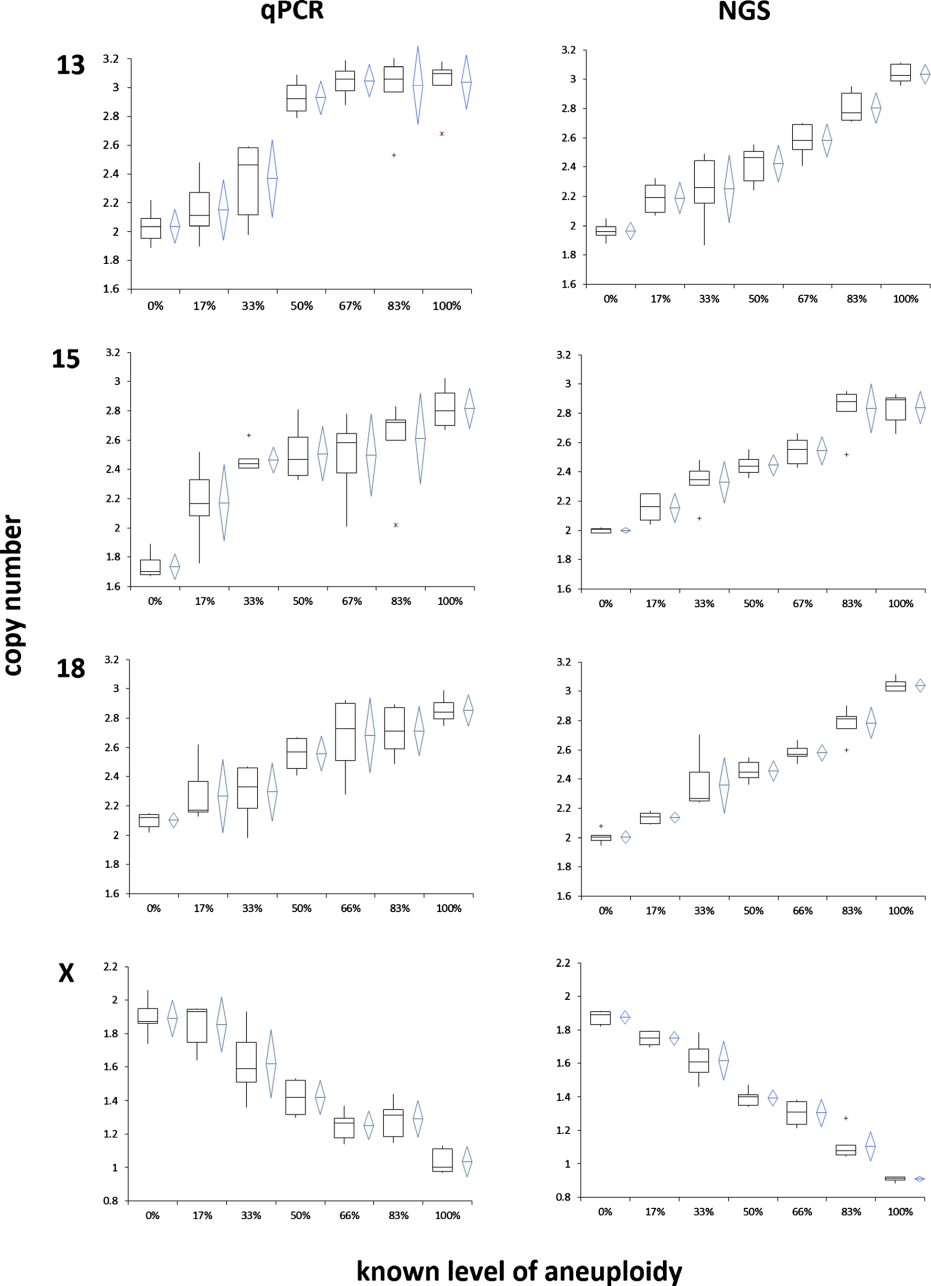
*Box* and *whisker* plots showing the distribution and variance of copy number assignments for target mosaic chromosomes as the percent of spike-in aneuploidy increases in the sample with each respective platform (qPCR and VeriSeq NGS). As the level of aneuploidy increases in the sample, there in an overall increase in the copy number of the chromosomes of interest (13, 15, and 18) and a decrease in the copy number of X as the percentage of female cells decreases in the sample

In order to illustrate the performance of each platform, example copy number plots for qPCR and NGS are shown in Fig. 3, which show the expected gradual change as the level of aneuploidy increases in each mixture. Reproducibility was considered by evaluating the distributions of copy number assignments for all replicates for both platforms and are shown in Fig. 4.

## Conclusions

Considerable attention has been given to the ability of contemporary CCS platforms to detect mosaicism. There are many factors to consider [17, 18], including predictive value of the biopsy for the remaining embryo and for actual clinical outcomes, the limits of detection when mosaicism is present within an individual trophectoderm biopsy, the developmental fate of different diploid/aneuploid compositions, and the chromosome specific and monosomy/trisomy-specific impact on development. This study focuses on the following limits of detection: the percentage of cells within a multicell sample that need to be aneuploid to allow detection, how often a platform can detect the abnormal cells, and how often artifacts of the technology result in incorrectly predicted abnormalities. The design was based upon the fundamental concept of evaluating preclinical validity with positive control cell lines. A similar strategy was key to the initial development of an accurate method of qPCR-based CCS for uniform aneuploidy [16]. While this method was initially designed with the intention of predicting constitutive aneuploidies, the ability to detect aneuploidy in a mosaic sample was not reported. Despite unsupported claims that NGS may provide superior capability to detect mosaicism [13], this study indicates equivalent performance when comparing NGS and qPCR, head to head, in a randomized blinded fashion. That is, there was not a significant difference in sensitivity or specificity of NGS and qPCR for detecting aneuploidy in mosaic samples. However, given the low copy number variance and linear response of NGS testing compared to qPCR (Fig. 4), further manual examination of itterations of NGS criteria could allow for improved detection of mosaicism. This linear response is similar to what is observed when a theoretical copy number contribution scale is created, and illustrates the great potential for these methodologies to accurately identify mosaic samples.

It is possible, however, that NGS may provide additional untapped information allowing for development of more sensitive methods of analysis beyond the default settings. While many groups have presented preliminary evidence for clinical predictive value, in some cases, criteria for designating an embryo as mosaic have not been defined. One recent study by Vera-Rodriguez et al. [14] described new criteria for predicting mosaicism in trophectoderm biopsies. However, although increased sensitivity was gained when applied to the present dataset, significant concominate loss in specificity (33 % reduction) was observed. This study by Vera-Rodriguez et al. is an important first step into establishing criteria for mosaicism and demonstrates the need to further evaluate a method and its ability to accurately predict aneuploidy in a mosaic sample through the use of cell lines. Greco et al. [11] also recently applied a custom algorithm to predict mosaicism within trophectoderm biopsies using aCGH data. Interestingly, when applied to clinical trophectoderm biopsies, 33 % of the embryos predicted as mosaic led to an apparent healthy live birth. This important observation emphasizes the fact that the clinical significance and impact of mosaicism on the ability of an embryo to produce healthy children presently remains unknown and also indicates that observations which may be consistent with mosaicism in the preimplantation embryo may not always be accurate. While the authors elected to attribute the poor predictive value to biological mechanisms of self-correction of diploid-aneuploid mosaics [19], or that these embryos were actually uniformly euploid to begin with, and the mosaic call was simply a technical artifact.

Defining the sensitivity and specificity of an assay is typically a prerequisite to clinical application. It is also important to establish before attempts are made at determining the overall prevalence of the abnormality, as methods which produce many false positives may significantly overestimate the overall frequency. This is particularly true with respect to mosaicism prevalence estimation. In fact, when strict criteria are used, such as observing trisomy and monosomy of the same chromosome within multiple biopsies from the same embryo (reciprocal aneuploidies), the rate of overall mosaicism prevalence is only ∼6 % [6, 7, 9, 20].

Beyond the sensitivity and specificity of detection within a mosaic sample, many other aspects of mosaicism may factor into the predictive value of a trophectoderm biopsy for the actual clinical outcome, including the distribution of aneuploidy in the remaining embryo, the level of aneuploidy present, and which chromosome is involved. Additional preclinical testing should include evaluating multiple biopsies of the same embryo in order to establish the predictive value of a single biopsy for the remaining embryo (i.e., true-positive rate). A prospective, blinded, non-selection study (as described in Scott et al. [21]) should be performed to establish positive and negative predictive values of a diagnosis for actual clinical outcomes [21, 22]. Finally, new clinical interventions should work toward randomized clinical trials ultimately to establish the efficacy of a diagnosis of mosaicism as a predictor of reproductive outcome and ongoing treatment regimes [2–4].
